# Association of COVID-19 Infection With Incident Diabetes

**DOI:** 10.1001/jamanetworkopen.2023.8866

**Published:** 2023-04-18

**Authors:** Zaeema Naveed, Héctor A. Velásquez García, Stanley Wong, James Wilton, Geoffrey McKee, Bushra Mahmood, Mawuena Binka, Drona Rasali, Naveed Z. Janjua

**Affiliations:** 1 British Columbia Centre for Disease Control, Vancouver, British Columbia, Canada; 2 School of Population and Public Health, University of British Columbia, Vancouver, British Columbia, Canada; 3 Department of Medicine, University of British Columbia, Vancouver, British Columbia, Canada; 4 Centre for Health Evaluation and Outcome Sciences, St Paul’s Hospital, Vancouver, British Columbia, Canada

## Abstract

**Importance:**

SARS-CoV-2 infection may lead to acute and chronic sequelae. Emerging evidence suggests a higher risk of diabetes after infection, but population-based evidence is still sparse.

**Objective:**

To evaluate the association between COVID-19 infection, including severity of infection, and risk of diabetes.

**Design, Setting, and Participants:**

This population-based cohort study was conducted in British Columbia, Canada, from January 1, 2020, to December 31, 2021, using the British Columbia COVID-19 Cohort, a surveillance platform that integrates COVID-19 data with population-based registries and administrative data sets. Individuals tested for SARS-CoV-2 by real-time reverse transcription-polymerase chain reaction (RT-PCR) were included. Those who tested positive for SARS-CoV-2 (ie, those who were exposed) were matched on sex, age, and collection date of RT-PCR test at a 1:4 ratio to those who tested negative (ie, those who were unexposed). Analysis was conducted January 14, 2022, to January 19, 2023.

**Exposure:**

SARS-CoV-2 infection.

**Main Outcomes and Measures:**

The primary outcome was incident diabetes (insulin dependent or not insulin dependent) identified more than 30 days after the specimen collection date for the SARS-CoV-2 test with a validated algorithm based on medical visits, hospitalization records, chronic disease registry, and prescription drugs for diabetes management. Multivariable Cox proportional hazard modeling was performed to evaluate the association between SARS-CoV-2 infection and diabetes risk. Stratified analyses were performed to assess the interaction of SARS-CoV-2 infection with diabetes risk by sex, age, and vaccination status.

**Results:**

Among 629 935 individuals (median [IQR] age, 32 [25.0-42.0] years; 322 565 females [51.2%]) tested for SARS-CoV-2 in the analytic sample, 125 987 individuals were exposed and 503 948 individuals were unexposed. During the median (IQR) follow-up of 257 (102-356) days, events of incident diabetes were observed among 608 individuals who were exposed (0.5%) and 1864 individuals who were not exposed (0.4%). The incident diabetes rate per 100 000 person-years was significantly higher in the exposed vs nonexposed group (672.2 incidents; 95% CI, 618.7-725.6 incidents vs 508.7 incidents; 95% CI, 485.6-531.8 incidents; *P* < .001). The risk of incident diabetes was also higher in the exposed group (hazard ratio [HR], 1.17; 95% CI, 1.06-1.28) and among males (adjusted HR, 1.22; 95% CI, 1.06-1.40). The risk of diabetes was higher among people with severe disease vs those without COVID-19, including individuals admitted to the intensive care unit (HR, 3.29; 95% CI, 1.98-5.48) or hospital (HR, 2.42; 95% CI, 1.87-3.15). The fraction of incident diabetes cases attributable to SARS-CoV-2 infection was 3.41% (95% CI, 1.20%-5.61%) overall and 4.75% (95% CI, 1.30%-8.20%) among males.

**Conclusions and Relevance:**

In this cohort study, SARS-CoV-2 infection was associated with a higher risk of diabetes and may have contributed to a 3% to 5% excess burden of diabetes at a population level.

## Key Points

**Question:**

Is SARS-Cov-2 infection associated with incident diabetes?

**Findings:**

In this cohort study of 629 935 individuals tested for SARS-CoV-2, the hazard of incident diabetes was significantly higher among individuals who tested positive for SARS-CoV-2 infection than those who tested negative. The fraction of incident diabetes cases attributable to SARS-CoV-2 infection was 3% to 5%.

**Meaning:**

This study found that SARS-CoV-2 infection was associated with a higher risk of diabetes, suggesting that these infections may have contributed to an excess burden of diabetes at the population level.

## Teaser

This cohort study investigates the association of SARS-CoV-2 infection with risk of incident diabetes among individuals in Canada.

## Introduction

Since its emergence in December 2019, SARS-CoV-2 has caused significant morbidity and mortality.^1^ While initial public health measures and research focused on understanding transmission, disease severity, prevention interventions, and treatment of acute infection, efforts to better understand the long-term sequelae and wider consequences of COVID-19 are now at the forefront. While acute infection primarily affects the respiratory system, other organ systems may also be involved, leading to various acute and chronic sequelae.^2^

Among other long-term sequelae of SARS-CoV-2 infection, emerging evidence suggests that COVID-19 may be associated with changes in the pathophysiology of diabetes.^3,4^ Diabetes has already been established as a risk factor associated with more severe COVID-19 respiratory outcomes, and SARS-CoV-2 infection was associated with the worsening of preexisting diabetes symptoms.^5,6,7,8^ However, it is not fully known if SARS-CoV-2 infection is associated with transient hyperglycemia during active infection or if metabolic alterations persist, associated with increased risk of subsequent diabetes among individuals with infections.

Current data on the association between SARS-CoV-2 infection and incident diabetes are sparse, and most published studies have been conducted with relatively small samples or have limitations related to participant selection or outcome ascertainment.^9,10,11,12,13^ Studies conducted to date have mostly identified a small but higher rate of incident diabetes among people with COVID-19 compared with those without COVID-19. To improve the understanding of this association between SARS-CoV-2 infection and incident diabetes, we conducted a large-scale, population-based cohort study using population-based registries and data sets with a comprehensive definition of incident diabetes and evaluated the potential association between COVID-19 infection, including severity of infection, and diabetes incidence. Finally, we calculated the population-attributable fraction (PAF) while controlling for potential confounders to estimate the population-level burden associated with SARS-CoV-2 infection.

## Methods

This cohort study followed the Strengthening the Reporting of Observational Studies in Epidemiology (STROBE) reporting guideline. The Behavioral Research Ethics Board at the University of British Columbia reviewed and approved this study. The data included in the cohort were collected as part of routine health care encounters, and deidentified data were used for analysis, so informed consent was not needed, per the Canadian Tri-Council Policy Statement.^14^

### Data Source

The study used data from the British Columbia COVID-19 Cohort, a public health surveillance platform integrating COVID-19 data sets (including testing, case, hospitalization, and vaccination data) with registry and administrative data holdings for the British Columbia population, including data on medical visits, hospitalizations, emergency department visits, prescription drug dispensations, chronic conditions, and vital statistics. See eTable 1 in Supplement 1 for more information on integrated data sets. The British Columbia COVID-19 Cohort was established as a public health surveillance system under the British Columbia Centre for Disease Control public health mandate.^15^

### Study Design and Population

We created a matched cohort of individuals tested for SARS-CoV-2 by real-time reverse transcription-polymerase chain reaction (RT-PCR) in British Columbia from January 1, 2020, to December 31, 2021. We excluded individuals with a history of diabetes prior to their RT-PCR test, as well as individuals who were identified to have diabetes or who died within 30 days of their positive RT-PCR test. We also excluded people residing in long-term care facilities given that they differ greatly from the general population in number and severity of comorbidities and level of health care access.

Eligible adult participants (aged ≥18 years) who tested positive for SARS-CoV-2 (ie, individuals who were exposed) were matched on sex (exact matching), age (3 years older or younger), and collection date of RT-PCR test (7 days before or after) at a 1:4 ratio with individuals who tested negative (ie, individuals who were unexposed). The collection date for the first positive test was considered for individuals with multiple positive tests. Individuals considered unexposed were those with no record of a positive RT-PCR test (ie, RT-PCR was negative for them). A single test was randomly selected for individuals who were unexposed and had multiple negative tests.

### Exposures and Outcomes

Our primary exposure of interest was RT-PCR–confirmed SARS-CoV-2 infection. The outcome of interest was incident diabetes (insulin dependent or not insulin dependent) identified more than 30 days after the index date (ie, the collection date for a positive COVID-19 test). The validated algorithm was based on medical visits, hospitalization records, chronic disease registry data, and prescription drug use for diabetes management (eTable 2 in Supplement 1).

### Covariate Measurement

In addition to sociodemographic matching variables (sex, age, and collection date) and exposure (SARS-CoV-2 infection), we examined a range of comorbidities potentially associated with the outcome to characterize our study population. Chronic conditions included acute myocardial infarction, asthma, chronic kidney disease, chronic liver disease, chronic obstructive pulmonary disease, depression, hypertension, glucocorticoid use, alcohol misuse, and injection drug use. Additionally, we adjusted for vaccination status and used material deprivation index^16^ (expressed as quintiles and measured at the dissemination-area geographic level) to account for socioeconomic status. See eTable 2 in Supplement 1 for more information on variable definitions and data sources.

### Statistical Analysis

Baseline characteristics of individuals in the matched cohort were compared between exposed and unexposed populations, represented as No. (%) and median (IQR) for categorical and continuous variables, respectively. Mann-Whitney *U* tests were performed for continuous variables and χ^2^ tests for categorical variables.

We imputed material deprivation values (missing for 40 043 of 629 935 individuals [6.4%]) based on geographic area (from the British Columbia Health Authority), age, exposure, and sex. Imputation was performed via linear discriminant analysis using the miceFast R library.^17^

Matched cohort study participants were followed up from 30 days after the index date (ie, the collection date of the RT-PCR test) to the earliest of identification of the outcome, death from any cause, or study end date (January 31, 2022). The number of incident diabetes events and person-days were determined for each group and subsequently used to calculate incidence rates of diabetes (number of events/100 000 person-years). Cumulative incidence curves were generated through the Kaplan-Meier method. Primarily, Cox proportional hazards regression models accounting for matched data were used to compare the risk of diabetes by exposure status for all participants.^18^ Separate subgroup analyses using Cox proportional hazards regression models were conducted by restricting the exposed group to individuals who were hospitalized and those who were admitted to an intensive care unit (ICU) to assess the risk of diabetes by disease severity. Interaction terms and likelihood ratio tests in the resulting nested models were used to assess interaction of SARS-CoV-2 infection with diabetes risk by sex, age, and vaccination status; stratified analyses were performed when evidence of such interaction was present. The confounder-adjusted population-attributable fraction was computed from Cox models.^19^ The model-based estimation of confounder-adjusted attributable fractions method does not allow the use of stratified Cox models. Hence, the attributable fraction in the general population was computed using a multivariable Cox model, including in a regular manner terms with evidence of nonproportionality. Additionally, multiple sensitivity analyses were conducted. We repeated analyses after excluding observations with imputed values from the analytic sample, including death as a competing risk, assessing the outcome separately as insulin-dependent and non–insulin-dependent diabetes, excluding individuals with less than 90 days of observation time to allow longer follow-up intervals for those who entered later in the study, and stratifying the sample by vaccination status.

The proportional hazards assumption was evaluated using statistical tests and scaled Schoenfeld residuals graphical diagnostics.^20,21^ When the proportional hazards assumption was violated for any factor other than the exposure of interest, the Cox model was stratified by the covariate not satisfying the assumption. Analyses were performed with R statistical software version 4.0.2 (R Project for Statistical Computing).^22^ The threshold for statistical significance was a 2-tailed *P* < .05. Analysis was conducted January 14, 2022, to January 19, 2023.

## Results

### Participant Profile

There were 210 677 individuals who tested positive for SARS-CoV-2 available for matching, with 1 396 245 individuals testing negative identified during the evaluation time. After initial matching of exposure groups, the preliminary sample comprised 186 138 individuals who were exposed and 744 552 individuals who were unexposed. After individuals with prevalent diabetes and those residing at long-term care facilities were excluded, the analytic sample consisted of 629 935 individuals (median [IQR] age, 32 [25.0-42.0] years; 322 565 females [51.2%]), including 125 987 individuals who were exposed and 503 948 individuals who were unexposed. Baseline characteristics of matched participants are shown in Table 1. A greater percentage of individuals who were exposed (28 006 individuals [22.2%]) were in the highest material deprivation quintile compared with those who were unexposed (65 349 individuals [13.0%]). Similarly, a greater proportion of individuals who were exposed were not vaccinated at the time of sample collection (95 009 individuals [75.4%]) than individuals who were unexposed (318 529 individuals [63.2%]).

**Table 1.** Distribution of Characteristics in Matched Cohort

Characteristic	Individuals, No. (%)		
	Unexposed (n = 503 948)	Exposed (n = 125 987)	Overall (N = 629 935)
Sex			
Female	258 052 (51.2)	64 513 (51.2)	322 565 (51.2)
Male	245 896 (48.8)	61 474 (48.8)	307 370 (48.8)
Age, median (IQR), y	32.0 (24.041.0)	34.0 (27.044.0)	32.0 (25.042.0)
Age group, y			
18-39	360 026 (71.4)	81 472 (64.7)	441 498 (70.1)
40-59	126 090 (25.0)	37 743 (30.0)	163 833 (26.0)
60-79	17 446 (3.5)	6593 (5.2)	24 039 (3.8)
≥80	386 (0.1)	179 (0.1)	565 (0.1)
Health authority			
Fraser	175 (0.1)	62 (0.1)	237 (0.1)
Interior	214 788 (42.6)	60 361 (47.9)	275 149 (43.7)
Northern	71 846 (14.2)	18 684 (14.8)	90 530 (14.4)
Vancouver Coastal	19 573 (3.9)	8997 (7.1)	28 570 (4.5)
Vancouver Island	128 079 (25.4)	29 400 (23.3)	157 479 (25.0)
Unknown or missing	69 487 (13.8)	8483 (6.7)	77 970 (12.4)
Diabetes incident event	1864 (0.4)	608 (0.5)	2472 (0.4)
Diabetes incidence rate (95% CI), per 100 000 person-days	508.7 (485.6-531.8)	672.2 (618.7-725.6)	541.1 (519.8-562.4)
Follow-up time, median (IQR), d	257 (102-357)	255 (99.0-354)	257(102-356)
History of acute myocardial infarction	2506 (0.5)	828 (0.7)	3334 (0.5)
Asthma	72 357 (14.4)	16 420 (13.0)	88 777 (14.1)
Chronic kidney disease	6066 (1.2)	1813 (1.4)	7879 (1.3)
Chronic liver disease	1187 (0.2)	319 (0.3)	1506 (0.2)
Chronic obstructive pulmonary disease	3710 (0.7)	1027 (0.8)	4737 (0.8)
Depression	130 556 (25.9)	31 262 (24.8)	161 818 (25.7)
Hypertension	26 909 (5.3)	8956 (7.1)	35 865 (5.7)
Alcohol misuse	16 574 (3.3)	4981 (4.0)	21 555 (3.4)
Injection drug use	22 389 (4.4)	6628 (5.3)	29 017 (4.6)
Glucocorticoids use	18 920 (3.8)	4841 (3.8)	23 761 (3.8)
Vaccination status			
Not vaccinated	318 529 (63.2)	95 009 (75.4)	413 538 (65.6)
Partially vaccinated	32 766 (6.5)	6841 (5.4)	39 607 (6.3)
Vaccinated	152 653 (30.3)	24 137 (19.2)	176 790 (28.1)
Material deprivation index score			
1 (Most privileged)	105 965 (21.0)	22 269 (17.7)	128 234 (20.4)
2	113 106 (22.4)	23 941 (19.0)	137 047 (21.8)
3	97 997 (19.4)	22 430 (17.8)	120 427 (19.1)
4	89 318 (17.7)	24 419 (19.4)	113 737 (18.1)
5 (Most deprived)	65 349 (13.0)	25 098 (19.9)	90 447 (14.4)
Unknown or missing	32 213 (6.4)	7830 (6.2)	40 043 (6.4)
Material deprivation index score (imputed)			
1 (Most privileged)	110 821 (22.0)	22 269 (17.7)	133 090 (21.1)
2	140 463 (27.9)	28 863 (22.9)	169 326 (26.9)
3	97 997 (19.4)	22 430 (17.8)	120 427 (19.1)
4	89 318 (17.7)	24 419 (19.4)	113 737 (18.1)
5 (Most deprived)	65 349 (13.0)	28 006 (22.2)	93 355 (14.8)

Study participants were followed up for a median (IQR) of 257 (102-356) days. During follow-up, events of incident diabetes were recorded among a total of 2472 individuals (0.4%), including 608 individuals who were exposed (0.5%) and 1864 individuals who were unexposed (0.4%). Among those diagnosed with incident diabetes, there were 1393 females (56.4%) and 1079 males (43.65%). The diabetes incidence rate per 100 000 person-years was significantly higher in the exposed group (672.2 incidents; 95% CI, 618.7-725.6 incidents) compared with the unexposed group (508.7 incidents; 95% CI, 485.6-531.8 incidents; *P* < .001) (Table 1). Descriptive characteristics of individuals with incident diabetes by SARS-CoV-2 status are shown in eTable 3 in Supplement 1. Overall and sex-specific cumulative incidence plots (Figure) stratified by exposure status exhibited a higher incidence over time in the exposed group.

**Figure.**
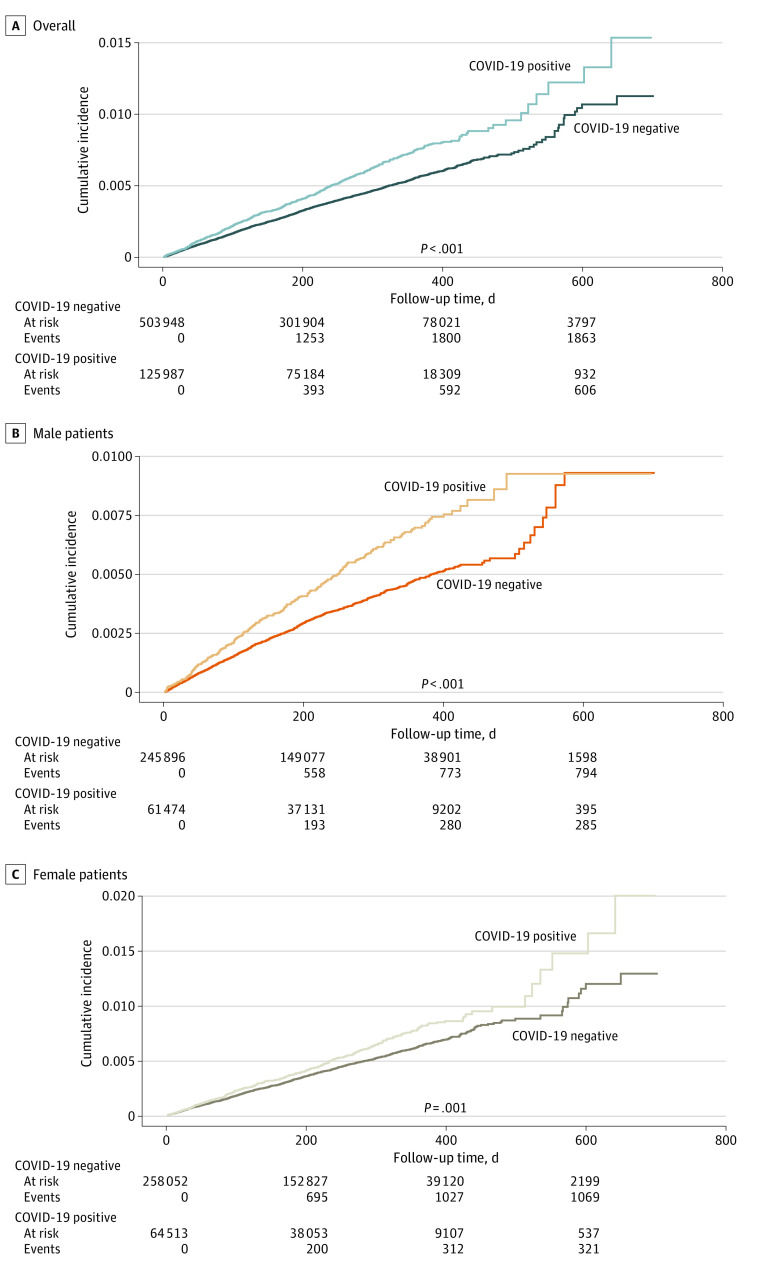
Cumulative Incidence of Incident Diabetes

### Association of SARS-CoV-2 Infection and Diabetes

The results of Cox proportional hazards models with and without COVID-19 severity subgrouping are shown in Table 2. The overall adjusted hazard ratio (HR) of incident diabetes was 1.17 (95% CI, 1.06-1.28) for individuals in the exposed vs unexposed group. There was an interaction between infection status and the association of sex with diabetes risk. Among males, SARS-CoV-2 infection was associated with significantly higher risk of incident diabetes (adjusted HR [aHR], 1.22; 95% CI, 1.06-1.40), while among females, results did not reach statistical significance (aHR, 1.12; 95% CI, 0.99-1.27). However, on further examination by COVID-19 severity, the overall and sex-specific adjusted risk of incident diabetes was higher among people with more severe disease, including individuals admitted to the ICU (HR, 3.29; 95% CI, 1.98-5.48) or hospital (HR, 2.42; 95% CI, 1.87-3.15). Additionally, there was an association among females, although the HRs were higher in males. Full models are shown in eTables 4 through 12 in Supplement 1.

**Table 2.** Hazard of Incident Diabetes After COVID-19 vs Unexposed Group

Population	Adjusted HR (95% CI)^a^		
	All participants	Hospitalized individuals	Individuals admitted to intensive care
Overall	1.17 (1.06-1.28)^b^	2.42 (1.87-3.15)^c^	3.29 (1.98-5.48)^d^
Males	1.22 (1.06-1.40)	2.84 (2.01-4.03)	3.74 (1.97-7.07)
Females	1.12 (0.99-1.27)^e^	1.94 (1.30-2.88)^e^	2.71 (1.18-6.18)^e^

^a^ Cox models were adjusted for age, sex (overall), acute myocardial infarction, asthma, chronic kidney disease, depression, hypertension, glucocorticoid use, vaccination status, and material deprivation index score.

^b^ Model stratified on age, sex, and material deprivation index score.

^c^ Model stratified on sex and depression.

^d^ Model stratified on sex.

^e^ Model stratified on material deprivation index score.

The confounder-adjusted population-attributable fraction indicated that overall 3.41% (95% CI, 1.20%-5.61%) of incident diabetes cases in our sample were attributable to SARS-CoV-2 infection; this percentage was 4.75% (95% CI, 1.30%-8.20%) among males (eTable 13 in Supplement 1). Nonproportionality issues did not seem consequential given that estimates for the exposure of interest from the nonstratified Cox model (aHR, 1.17; 95% CI, 1.06-1.28) were similar to those obtained with the stratified Cox model (aHR, 1.16; 95% CI, 1.06-1.28).

In the sensitivity analysis subclassifying diabetes as insulin dependent and not insulin dependent, 784 and 1688 events were observed, respectively. Cox proportional hazards modeling showed an association between COVID-19 exposure and non–insulin-dependent diabetes only (HR, 1.17; 95% CI, 1.04-1.31) (eTables 14-15 in Supplement 1). The sensitivity analysis excluding individuals with less than 90 days of observation time showed the same pattern as the primary analysis (eTables 16-18 in Supplement 1). Finally, the vaccine-stratified analysis indicated an association between COVID-19 exposure and diabetes in the unvaccinated group, while no association was observed in the remaining vaccination status strata (partial and fully vaccinated) (eTables 19-21 in Supplement 1).

## Discussion

In this population-based cohort study, we observed a statistically significantly higher risk of incident diabetes among individuals with SARS-CoV-2 infection compared with those without infection overall and specifically among males. We also found higher risk of diabetes among individuals with more severe disease in males and females. These findings suggest that COVID-19 infection may continue to be associated with outcomes in organ systems involved in regulating blood glucose in the postacute phase and so may have contributed to the 3% to 5% of excess incident diabetes cases found in this study. These findings suggest that SARS-CoV-2 infection may be associated with a higher burden of diabetes at the population level.

Our overall results were consistent with several other studies finding higher risk of incident diabetes after of SARS-CoV-2 infection; however, the increase in risk was lower in our analysis compared with other studies. Xie et al^23^ used national databases of the US Department of Veterans Affairs (VA) and reported a higher risk of incident diabetes among 30-day survivors of COVID-19 compared with individuals in the control group over a median follow-up of 352 days (HR, 1.40; 95% CI, 1.36-1.44). The VA population in this study comprised predominantly males with a higher median age than the general population, while our study population was demographically diverse across age and sex. Furthermore, compared with the general US population, the VA is underrepresented in Asian and Hispanic or Latino patients and overrepresented in Black and non-Hispanic White populations, while our study population was drawn from the general population. In addition, an increased overweight and obesity prevalence in the US population (23% of individuals with obesity in British Columbia vs 41% in the VA) may also be associated with these differences.^24,25^

Disregarding the severity of COVID-19, we found that the association between SARS-CoV-2 infection and diabetes risk was significant only for males, which could be attributed to sex-specific immune responses, such as higher SARS-CoV-2 IgG antibody serum concentrations in females compared with males.^26^ However, by restricting the sample to individuals with a COVID-19 hospitalization and ICU admission, the increase in risk was not only greater for higher-severity groups, but also significant for males and females. Previous studies had mixed results. Similar to our finding, Wander et al^27^ used US VA data to find that SARS-CoV-2 was associated with higher odds of incident diabetes (mean follow-up, 193 days) among males (odds ratio, 1.95; 95% CI, 1.80-2.12) but not females. However, in contrast to our findings, Wander et al^27^ observed the same results when they restricted the sample to individuals hospitalized within 30 days after the index date, with an association among males and not females. Use of dissimilar comparison groups may account for the contrasting finding given that Wander et al^27^ restricted the COVID-19–negative group to hospitalized individuals, while we did not. Furthermore, adjustment of different confounders in the 2 studies and population demographic differences (the VA has an older and predominantly male participant population with lower income and socioeconomic status levels) may be associated with the diverse findings.

Xie et al^23^ used the same database and reported a higher hazard of diabetes at 12 months for individuals infected with SARS-CoV-2 irrespective of severity within males (HR, 1.37; 95% CI, 1.33-1.41) and females (HR, 1.28; 95% CI, 1.17-1.40). Although Xie et al^23^ did not stratify by sex when conducting severity-specific analyses, they observed that COVID-19 infection was positively associated with risks and burdens of postacute incident diabetes, with the increase in risk graded by severity of COVID-19. A similar study by Al-Aly et al^28^ demonstrated a positive diabetes risk gradient across the care setting of acute COVID-19 infection from individuals who were not hospitalized to those who were hospitalized, and risk was highest in patients who were admitted to ICUs. However, the authors did not conduct any sex-specific analysis. Daugherty et al^10^ conducted a retrospective cohort study among commercially insured adults in the US to investigate the association between subacute SARS-CoV-2 infection and a wide range of clinical sequelae, reporting a higher hazard of incident non–insulin-dependent diabetes among individuals infected with SARS-CoV-2 (HR, 2.47; 95% CI, 1.14-5.38) over a 6-month follow-up. Daugherty et al^10^ further found that significant excess risk for incident non–insulin-dependent diabetes after acute COVID-19 did not differ between men and women. The disagreement may be associated with the specificity of the outcome, differences in study populations, or other methodological differences.

Differentiating between type of diabetes (insulin dependent and not insulin dependent), COVID-19 was associated only with non–insulin-dependent disease. However, we may be limited by the small number of insulin-dependent diabetes events in our sample. Regarding this finding, we are not able to draw a conclusive comparison with previous studies given that they classified diabetes as type 1 or type 2 based on single *International Statistical Classification of Diseases and Related Health Problems, Tenth Revision *(*ICD-10*) codes. Al-Aly et al^28^ used VA data and found that COVID-19 was associated with an increased risk of type 2 diabetes only. In contrast, a large-scale retrospective study conducted by Qeadan et al^29^ using Cerner Real-World Data from the US found significantly higher odds of developing new-onset type 1 diabetes in patients with COVID-19 diagnosis (odds ratio, 1.42; 95% CI, 1.38-1.46) compared with those without COVID-19. However, that study included participants without any age-based exclusion, whereas our study population included only adults. Rathmann et al^9^ used a national-level outpatient primary care data set from Germany and reported a higher incidence rate of type 2 diabetes among individuals with COVID-19 compared with the control group (15.8 vs 12.3 incidents/1000 person-years) and no association for other forms of diabetes. Similarly, Birabaharan et al^30^ found a higher risk of type 2 diabetes among people with mild COVID-19 (relative risk, 1.54; 95% CI, 1.46-1.62) and moderate-to-severe COVID-19 (relative risk, 1.46; 95% CI, 1.26-1.69) compared with those in control groups with mild and moderate-to-severe influenza, respectively.

The underlying pathophysiological mechanism explaining the association between COVID-19 infection and diabetes is not clearly understood. However, multiple interrelated processes may be responsible. It has been demonstrated that SARS-CoV-2 can directly infect human pancreatic β cells expressing angiotensin-converting enzyme 2 receptors and induce β cell apoptosis, impacting pancreatic insulin levels and secretion.^31^ This destruction of β cells is relevant to type 1 and type 2 diabetes. Furthermore, chronic low-grade inflammation resulting from proinflammatory pathways in sites such as adipose tissue may also play a role in the pathogenesis and progression of type 2 diabetes.^4^ We excluded any acute events (ie, within 30 days of the index date) to avoid capturing transient hyperglycemia that can result from acute inflammatory response, medication used for treatment (especially glucocorticoids), and stress (eg, cytokine storm).^32^

The major strength of our study was the comprehensive population-based capture of SARS-CoV-2 infection and integration of these data with population-based registries and health care–use databases used to identify incident diabetes and ascertain potential confounders. In addition to calculating HRs, we have also provided estimates of the population-attributable fraction that have not been presented before, to our knowledge. Additionally, compared with most previous studies, we had a longer follow-up time, enabling us to investigate the hazard of diabetes as a long-term sequela of COVID-19.

### Limitations

Certain limitations of the study are as follows: Although we matched and adjusted for potential confounders, we cannot rule out residual confounding or that, due to a lack of information, some covariates could have affected the association between COVID-19 and incident diabetes; these may include obesity status and physical activity level. We cannot completely rule out surveillance bias given that individuals diagnosed with COVID-19 may have been more likely to receive increased screening or testing compared with those in the COVID-19–negative comparison group. Similarly, although we attempted to account for health care access and use by adjusting for material deprivation index score, it is difficult to fully account for differential health care system use. Additionally, it is possible that some participants we classified as unexposed may have contracted COVID-19 before or after entering the study but were not tested or had false-negative results.

## Conclusions

This large, population-based cohort study was conducted with robust methods addressing limitations of previous studies that had limited generalizability (eg, using specific population groups, such as hospitalized individuals, commercially insured individuals, and veterans, who are predominantly male). We found that SARS-CoV-2 infection was associated with a higher risk of incident diabetes overall and among males and that severe disease was associated with a higher risk of diabetes among males and females. These results suggest that infection with SARS-CoV-2 may have contributed to a 3% to 5% excess burden of diabetes, which may be associated with a substantial number of diabetes cases with bearing on health care needs for the management of diabetes and its complications. Our study highlights the importance of health agencies and clinicians being aware of the potential long-term consequences of COVID-19 and monitoring people after COVID-19 infection for new-onset diabetes for timely diagnosis and treatment.

## Notes

Supplement 1. **eTable 1.** Data Sets Integrated Within British Columbia COVID-19 Cohort
eReferences.
**eTable 2.** Definitions for Comorbidity Variables Derived From Administrative Data Sets **eTable 3.** Distribution of Characteristics Among Individuals With Incident Diabetes Cases by COVID-19 Exposure Status **eTable 4.** Overall Adjusted Hazard Ratios for Incident Diabetes From Cox Model Stratified by Age, Sex, and Material Deprivation Index **eTable 5.** Adjusted Hazard Ratios for Incident Diabetes in Males **eTable 6.** Adjusted Hazard Ratios for Incident Diabetes in Females From Cox Model Stratified by Material Deprivation Index Score **eTable 7.** Overall Adjusted Hazard Ratios for Incident Diabetes Among Individuals Hospitalized With COVID-19 vs Test-Negative Control Group From Cox Model Stratified by Sex and Depression Status **eTable 8.** Adjusted Hazard Ratios for Incident Diabetes Among Males Hospitalized With COVID-19 vs Test-Negative Control Group **eTable 9.** Adjusted Hazard Ratios for Incident Diabetes Among Females Hospitalized With COVID-19 vs Test-Negative Controls From Cox Model Stratified by Material Deprivation Index Score **eTable 10.** Overall Adjusted Hazard Ratios for Incident Diabetes Among Individuals Admitted to Intensive Care Unit With COVID-19 vs Test-Negative Control Group in Cox Model Stratified by Sex **eTable 11.** Adjusted Hazard Ratios for Incident Diabetes Among Males Admitted to Intensive Care Unit With COVID-19 vs Test-Negative Control Group **eTable 12.** Adjusted Hazard Ratios for Incident Diabetes Among Females Admitted to Intensive Care Unit With COVID-19 vs Test-Negative Control Group From Cox Model Stratified by Material Deprivation Index Score **eTable 13.** Estimated Population-Attributable Fraction and 95% Wald CI **eTable 14.** Overall Adjusted Hazard Ratio for Incident Insulin-Dependent Diabetes From Cox Model Stratified by Age and Depression Status **eTable 15.** Overall Adjusted Hazard Ratio for Incident Non–Insulin-Dependent Diabetes From Cox Model Stratified by Sex **eTable 16.** Overall Adjusted Hazard Ratios for Incident Diabetes Among Individuals With More Than 90 d Observation Time From Cox Model Stratified by Sex **eTable 17.** Adjusted Hazard Ratios for Incident Diabetes Among Males With More Than 90 d Observation Time From Cox Model **eTable 18.** Adjusted Hazard Ratios for Incident Diabetes Among Females With More Than 90 d Observation Time From Cox Model **eTable 19.** Overall Adjusted Hazard Ratios for Incident Diabetes in Unvaccinated Subpopulation From Cox Model Stratified by Sex, Age, Material Deprivation Index Score **eTable 20.** Overall Adjusted Hazard Ratios for Incident Diabetes in Partially Vaccinated Subpopulation **eTable 21.** Overall Adjusted Hazard Ratios for Incident Diabetes in Vaccinated Subpopulation

Supplement 2.
Data Sharing Statement
