# Errors in Table 1

**DOI:** 10.1001/jamanetworkopen.2023.16822

**Published:** 2023-05-17

In the Original Investigation titled “Association of COVID-19 Infection With Incident Diabetes,”^1^ published April 18, 2023, there were errors in Table 1 for the “Health authority” variable. The data were incorrectly aligned, with the last category’s numbers listed in the first row and each subsequent category’s numbers listed 1 row too low. This article has been corrected.^1^
