# Supplementary material for: Association of COVID-19 Infection With Incident Diabetes
Source: JAMA Netw Open. 2023 Apr 18;6(4):e238866. doi: 10.1001/jamanetworkopen.2023.8866 (PMC10114057; doi:10.1001/jamanetworkopen.2023.8866)
Supplement: Supplement 1. — eTable 1. Data Sets Integrated Within British Columbia COVID-19 Cohort eReferences. eTable 2. Definitions for Comorbidity Variables Derived From Administrative Data Sets eTable 3. Distribution of Characteristics Among Individuals With Incident Diabetes Cases by COVID-19 Exposure Status eTable 4. Overall Adjusted Hazard Ratios for Incident Diabetes From Cox Model Stratified by Age, Sex, and Material Deprivation Index eTable 5. Adjusted Hazard Ratios for Incident Diabetes in Males eTable 6. Adjusted Hazard Ratios for Incident Diabetes in Females From Cox Model Stratified by Material Deprivation Index Score eTable 7. Overall Adjusted Hazard Ratios for Incident Diabetes Among Individuals Hospitalized With COVID-19 vs Test-Negative Control Group From Cox Model Stratified by Sex and Depression Status eTable 8. Adjusted Hazard Ratios for Incident Diabetes Among Males Hospitalized With COVID-19 vs Test-Negative Control Group eTable 9. Adjusted Hazard Ratios for Incident Diabetes Among Females Hospitalized With COVID-19 vs Test-Negative Controls From Cox Model Stratified by Material Deprivation Index Score eTable 10. Overall Adjusted Hazard Ratios for Incident Diabetes Among Individuals Admitted to Intensive Care Unit With COVID-19 vs Test-Negative Control Group in Cox Model Stratified by Sex eTable 11. Adjusted Hazard Ratios for Incident Diabetes Among Males Admitted to Intensive Care Unit With COVID-19 vs Test-Negative Control Group eTable 12. Adjusted Hazard Ratios for Incident Diabetes Among Females Admitted to Intensive Care Unit With COVID-19 vs Test-Negative Control Group From Cox Model Stratified by Material Deprivation Index Score eTable 13. Estimated Population-Attributable Fraction and 95% Wald CI eTable 14. Overall Adjusted Hazard Ratio for Incident Insulin-Dependent Diabetes From Cox Model Stratified by Age and Depression Status eTable 15. Overall Adjusted Hazard Ratio for Incident Non–Insulin-Dependent Diabetes From Cox Model Stratified by Sex eTable 16. Overal [file jamanetwopen-e238866-s001.pdf]

Supplemental Online Content

Naveed Z, Velásquez García HA, Wong S, et al. Association of COVID-19 Infection With Incident Diabetes. *JAMA Netw Open*. 2023;6(4):e238866. doi:10.1001/jamanetworkopen.2023.8866

eTable 1. Data Sets Integrated Within British Columbia COVID-19 Cohort

eReferences.

eTable 2. Definitions for Comorbidity Variables Derived From Administrative Data Sets

eTable 3. Distribution of Characteristics Among Individuals With Incident Diabetes Cases by COVID-19 Exposure Status

eTable 4. Overall Adjusted Hazard Ratios for Incident Diabetes From Cox Model Stratified by Age, Sex, and Material Deprivation Index

eTable 5. Adjusted Hazard Ratios for Incident Diabetes in Males

eTable 6. Adjusted Hazard Ratios for Incident Diabetes in Females From Cox Model Stratified by Material Deprivation Index Score

eTable 7. Overall Adjusted Hazard Ratios for Incident Diabetes Among Individuals Hospitalized With COVID-19 vs Test-Negative Control Group From Cox Model Stratified by Sex and Depression Status

eTable 8. Adjusted Hazard Ratios for Incident Diabetes Among Males Hospitalized With COVID-19 vs Test-Negative Control Group

eTable 9. Adjusted Hazard Ratios for Incident Diabetes Among Females Hospitalized With COVID-19 vs Test-Negative Controls From Cox Model Stratified by Material Deprivation Index Score

eTable 10. Overall Adjusted Hazard Ratios for Incident Diabetes Among Individuals Admitted to Intensive Care Unit With COVID-19 vs Test-Negative Control Group in Cox Model Stratified by Sex

eTable 11. Adjusted Hazard Ratios for Incident Diabetes Among Males Admitted to Intensive Care Unit With COVID-19 vs Test-Negative Control Group

eTable 12. Adjusted Hazard Ratios for Incident Diabetes Among Females Admitted to Intensive Care Unit With COVID-19 vs Test-Negative Control Group From Cox Model Stratified by Material Deprivation Index Score

eTable 13. Estimated Population-Attributable Fraction and 95% Wald CI

eTable 14. Overall Adjusted Hazard Ratio for Incident Insulin-Dependent Diabetes From Cox Model Stratified by Age and Depression Status

© 2023 Naveed Z et al. *JAMA Network Open.*

eTable 15. Overall Adjusted Hazard Ratio for Incident Non–Insulin-Dependent Diabetes From Cox Model Stratified by Sex

eTable 16. Overall Adjusted Hazard Ratios for Incident Diabetes Among Individuals With More Than 90 d Observation Time From Cox Model Stratified by Sex

eTable 17. Adjusted Hazard Ratios for Incident Diabetes Among Males With More Than 90 d Observation Time From Cox Model

eTable 18. Adjusted Hazard Ratios for Incident Diabetes Among Females With More Than 90 d Observation Time From Cox Model

eTable 19. Overall Adjusted Hazard Ratios for Incident Diabetes in Unvaccinated Subpopulation From Cox Model Stratified by Sex, Age, Material Deprivation Index Score

eTable 20. Overall Adjusted Hazard Ratios for Incident Diabetes in Partially Vaccinated Subpopulation

eTable 21. Overall Adjusted Hazard Ratios for Incident Diabetes in Vaccinated Subpopulation

This supplemental material has been provided by the authors to give readers additional information about their work.

eTable 1. Data Sets Integrated Within British Columbia COVID-19 Cohort

**British Columbia Centre for Disease Control (BCCDC), Provincial Heath Services Authority (PHSA) and Regional Health Authority data sources:**	**Data Date Ranges:**
Integrated COVID-19 laboratory dataset (SARS-CoV2 tests from private/public labs, includes sequencing and screening data) [S1]	Jan 2020-onward
COVID-19 surveillance case data (information collected on all probable/confirmed cases as part of public health follow up) [S2]	Jan 2020-onward
Provincial COVID-19 Monitoring Solution (critical and non-critical care hospital census data) [S3]	Jan 2020-onward
Provincial Immunizations Registry (COVID-19 vaccination data) [S4]	Dec 2020-onward
Provincial Laboratory Information Solution (laboratory tests from private/public labs) [S5]	Jan 2020-onward
Public Health Reporting Data warehouse (Influenza laboratory tests) [S6]	Jan 2008-onward
Emergency department visits (hospital-based and community-based ambulatory care)	Mar 2020-onward
**Ministry of Health (MoH) Administrative Data Sources:**	**Data Date Ranges:**
Client Roster (CR) (registry of enrollment in the universal public health insurance plan including residential history) [S7]	2008/9-onward
Discharge Abstracts Database (DAD) (hospital discharge records) [S8]	2008/9-onward
Medical Services Plan (MSP) (physician diagnostic and billing data for services provided through universal public health insurance plan) [S9]	2008/9-onward
PharmaNet (Pharma) (prescription drugs dispensed from community pharmacies, includes medications covered by public and private insurance plans) [S10]	2008/9-onward
BC Vital Statistics (VS) (deaths registry) [S11]	2008/9-onward
National Ambulatory Care Reporting System (NACRS) (hospital-based and community-based ambulatory care) [S12]	2011/12–onward
Chronic Disease Registry [S13]	2008/9-2018/19
811 Calls (respiratory calls only) [S14]	2014-onward
Health System Matrix [S15]	2018/19-onward
Population Grouper Methodology [S16]	2008/9-onward

© 2023 Naveed Z et al. *JAMA Network Open.*

eReferences

S1. British Columbia Centre for Disease Control [creator]. Integrated COVID-19 laboratory dataset (SARS-CoV2 tests from private/public labs). Public Health Reporting Data Warehouse, British Columbia Centre for Disease Control [publisher] (2021). 2022.

S2. British Columbia Centre for Disease Control [creator]. COVID-19 surveillance case data. British Columbia Centre for Disease Control [publisher]. (2021). 2022.

S3. Provincial Health Services Authority [creator]. Provincial COVID-19 Monitoring Solution. Provincial Health Services Authority [publisher]. (2021). 2022.

S4. Provincial Health Services Authority [creator]. Provincial Public Health Information Systems [publisher]. (2021). 2022.

S5. Provincial Health Services Authority [creator]. COVID-19 vaccination data. Provincial Immunizations Registry, Provincial Public Health Information Systems [publisher]. (2021). 2022.

S6. British Columbia Centre for Disease Control [creator]. Respiratory datamart, Public Health Reporting Data Warehouse, British Columbia Centre for Disease Control [publisher] (2021). 2022.

S7. British Columbia Ministry of Health [creator]. Client Roster (Client Registry System/Enterprise Master Patient Index). British Columbia Ministry of Health [publisher]. Data Extract. MOH (2021). 2022. https://www2.gov.bc.ca/gov/content/health/health-forms/online-services

S8. British Columbia Ministry of Health [creator]. Discharge Abstract Database (Hospital Separations). British Columbia Ministry of Health [publisher]. Data Extract. MOH (2021). 2022. https://www2.gov.bc.ca/gov/content/health/health-forms/online-services

S9. British Columbia Ministry of Health [creator]. Medical Services Plan (MSP) Payment Information File. British Columbia Ministry of Health [publisher]. Data Extract. MOH (2021). 2021.https://www2.gov.bc.ca/gov/content/health/health-forms/online-services

S10. British Columbia Ministry of Health [creator]. PharmaNet. British Columbia Ministry of Health [publisher]. Data Extract. MOH (2021). 2022. https://www2.gov.bc.ca/gov/content/health/health-forms/online-services

S11. BC Vital Statistics Agency [creator]. Vital Statistics Deaths. BC Vital Statistics Agency [publisher]. Data Extract. BC Vital Statistics Agency (2021). 2022. https://www2.gov.bc.ca/gov/content/health/health-forms/online-services

S12. British Columbia Ministry of Health [creator]. National Ambulatory Care Reporting System. British Columbia Ministry of Health [publisher]. Data Extract. MOH (2021). 2022. https://www2.gov.bc.ca/gov/content/health/health-forms/online-services

S13. British Columbia Ministry of Health [creator]. Chronic Disease Registry. British Columbia Ministry of Health [publisher]. Data Extract. MOH (2021). 2022. https://www2.gov.bc.ca/gov/content/health/health-forms/online-services

S14. British Columbia Ministry of Health [creator]. 811 calls. British Columbia Ministry of Health [publisher]. Data Extract. MOH (2021). 2022. https://www2.gov.bc.ca/gov/content/health/health-forms/online-services

S15. British Columbia Ministry of Health [creator]. Health System Matrix. British Columbia Ministry of Health [publisher]. Data Extract. MOH (2021). 2022. https://www2.gov.bc.ca/gov/content/health/health-forms/online-services

S16. British Columbia Ministry of Health [creator]. Population Grouper Methodology. British Columbia Ministry of Health [publisher]. Data Extract. MOH (2021). 2022. https://www2.gov.bc.ca/gov/content/health/health-forms/online-services

© 2023 Naveed Z et al. *JAMA Network Open.*

eTable 2. Definitions for Comorbidity Variables Derived From Administrative Data Sets

Element/variable	Data source	Definition	Reference
Matching variables			
Sex	COVID-19 surveillance case data (ccap_covid_case)	Categorized as male and female	British Columbia Centre for Disease Control [creator]. COVID-19 surveillance case data. British Columbia Centre for Disease Control [publisher]
Age	COVID-19 surveillance case data (ccap_covid_case)	Age in years at time of collection date. For analyses age was categorized as 18-39, 40-59, 60-67 and 80+	British Columbia Centre for Disease Control [creator]. COVID-19 surveillance case data. British Columbia Centre for Disease Control [publisher]
Collection date	COVID-19 surveillance case data (ccap_covid_case)	The sample collection date for the real-time reverse transcription–polymerase chain reaction (RT-PCR)	British Columbia Centre for Disease Control [creator]. COVID-19 surveillance case data. British Columbia Centre for Disease Control [publisher]
Outcome			
Diabetes mellitus (DM)	• Discharge Abstract Database (DAD)<br>• Medical Services Plan (MSP)<br>• Health Authority emergency department data (NACRS-HA)<br>• National Ambulatory Care Reporting System (NACRS-MOH)<br>• PharmaNet	Diabetes mellitus was defined as the occurrence of the second of 2 MSP, within one year, or 1 hospitalization diagnostic code for diabetes mellitus, or the prescription of at least two oral hypoglycemic drugs or insulin within one year. Physician Billing Data: MSP ICD-9 diagnostic codes: starting with 250.<br><br>Hospitalization Data: DAD1/ICD-9-CM: starting with 250.<br><br>DAD2/ICD-10-CA: starting with E10, E11, E12, E13 or E14.<br><br>PharmaNet Data: DIN PIN numbers: 5894, 6009, 12556, 12564, 12599, 12602, 12610, 13730, 13889, 15598, 21350, 21849, 24708, 24716, 93033, 156663, 156728, 178543, 209872, 209937, 237000, 244449, 271330, 274119, 274127, 275409, 275417, 275425, 312711, 312762, 314552, 377937, 399302, 420336, 430986, 431168, 446564, 446572, 446580,446599, 446602,	Jeong D, Karim ME, Wong S, Wilton J, Butt ZA, Binka M, Adu P, Bartlett S, Pearce M, Clementi E, Yu A, Alvarez M, Samji, Velasquez H, Abdia Y, Krajden M, Janjua NZ. (2021). Impact of HCV infection and ethnicity on incident type 2 diabetes: findings from a large population-based cohort in British Columbia. *BMJ Open Diabetes Research & Care*, 9(1):e002145, doi: 10.1136/bmjdrc-2021-002145

© 2023 Naveed Z et al. *JAMA Network Open.*

Element/variable	Data source	Definition	Reference
		446610, 454753, 480290, 480304, 513644, 514535, 514551, 539201,539244, 542911, 542938, 542946, 546348, 552259, 552267, 552275, 554820, 586714,586773, 587737, 612162, 612170, 612189, 612197, 612200, 612219, 612227, 612235,612243, 612251, 612278, 612359, 614416, 628301, 632651, 632678, 632686, 632694,644358, 646148, 648094, 650935, 720933, 720941, 723789, 733075, 765996, 773654,795879, 808733, 808741, 889091, 889105, 889113, 889121, 999717, 999814, 1900927,1900935, 1913654, 1913662, 1913670, 1913689, 1934066, 1934074, 1934082, 1934090,1934104, 1934112, 1959212, 1959220, 1959239, 1959352, 1959360, 1962639, 1962647, 1962655, 1962663, 1985930, 1985949, 1985957, 1985965, 1985973, 1985981, 1986085,1986791, 1986805, 1986813, 1986821, 1987534, 1987542, 1987828, 1987836, 2020734,2020742, 2022230, 2022249, 2024217, 2024225, 2024233, 2024241, 2024268, 2024276, 2024284, 2024292, 2024306, 2024314, 2024322, 2024403, 2024446, 2025248, 2025256,2045710, 2084341, 2085887, 2099233, 2147521, 2147548, 2148765, 2155850, 2162822,2162849, 2167786, 2188902, 2190885, 2190893, 2220628, 2223562, 2224550, 2224569, 2224771, 2224798, 2226804, 2226812, 2228920, 2228939, 2229516, 2229517, 2229519,2229595, 2229596, 2229656, 2229704, 2229705, 2229785, 2229994, 2230026, 2230027,2230036, 2230037,	

© 2023 Naveed Z et al. *JAMA Network Open.*

Element/variable	Data source	Definition	Reference
		2230443, 2230444, 2230475, 2230670, 2230671, 2231058, 2231095, 2231096, 2231389, 2233562, 2233999, 2234513, 2234514, 2236543, 2236548, 2236733,2236734, 2236985, 2236986, 2237531, 2238103, 2238469, 2238470, 2238471, 2238698,2238827, 2239081, 2239214, 2239474, 2239475, 2239476, 2239924, 2239925, 2239926, 2240294, 2240295, 2240297, 2241111, 2241112, 2241113, 2241114, 2241283, 2241310,2242095, 2242096, 2242572, 2242573, 2242574, 2242589, 2242726, 2242783, 2242793,2242794, 2242931, 2242974, 2242987, 2244353, 2245247, 2245272, 2245273, 2245274, 2245397, 2245438, 2245439, 2245440, 2245689, 2246820, 2246821, 2246964, 2246965,2247085, 2247086, 2247087, 2248008, 2248009, 2248210, 2248440, 2248441, 2248453,2251930, 2252945, 2252953, 2254719, 2257726, 2257734, 2258781, 2258803, 2258811, 2265435, 2265443, 2265575, 2265583, 2268493, 2268507, 2269031, 2269058, 2269589,2269597, 2269600, 2269619, 2271842, 2273101, 2273128, 2273136, 2273756, 2273764,2273772, 2274248, 2274256, 2274264, 2274272, 2274914, 2274922, 2274930, 2275864, 2275872, 2276410, 2279061, 2279088, 2279126, 2279460, 2279479, 2279487, 2284545,2284553, 2284782, 2284790, 2287072, 2294338, 2294346, 2294400, 2295377, 2295385,2295393, 2297795, 2297906, 2297914, 2297922, 2298279, 2298287, 2298295, 2300451, 2301423, 2301431, 2301458, 2302861, 2302888, 2302896, 2302942, 2302950, 2302977,2303124, 2303132,	

© 2023 Naveed Z et al. *JAMA Network Open.*

Element/variable	Data source	Definition	Reference
		2303140, 2303442, 2303450, 2303469, 2303922, 2305062, 2306166,2306174, 2306182, 2307170, 2307189, 2307197, 2307553, 2307561, 2307588, 2307634, 2307642, 2307650, 2307669, 2307677, 2307723, 2312050, 2312069, 2312077, 2313596,2314894, 2314908, 2316544, 2320754, 2320762, 2320770, 2321475, 2321483, 2321491,2326329, 2326337, 2326345, 2326477, 2326485, 2326493, 2331519, 2331527, 2333554, 2333856, 2333864, 2333872, 2334437, 2334445, 2336316, 2339110, 2339129, 2339587,2339595, 2340763, 2340771, 2341522, 2341603, 2343606, 2343614, 2345366, 2345374,2345382, 2345854, 2345862, 2348578, 2350459, 2350467, 2351056, 2351064, 2353377, 2353385, 2354144, 2354152, 2354160, 2354349, 2354357, 2354365, 2354926, 2354934, 2354942, 2355663, 2355671, 2355698, 2356422, 2357453, 2357461, 2357488, 2357887, 2357895, 2357909, 2357917, 2357925, 2361264, 2361272, 2361809, 2361817, 2363232, 2363240, 2363259, 2363518, 2363704, 2363712, 2364506, 2364514, 2365286, 2365294, 2365529, 2365537, 2366347, 2366355, 2366363, 2370921, 2373270, 2373289, 2373297, 2374013, 2374021, 2374048, 2374587, 2374595, 2375842, 2375850, 2375869, 2375877,2377209, 2378043, 2378051, 2378116, 2378124, 2378620, 2378639, 2378841, 2378868,2379767, 2379775, 2380196, 2380218, 2380722, 2380730, 2384906, 2384914, 2384922,2385341, 2385368, 2388766, 2388774, 2388839, 2388847, 2389169, 2389177, 2389185,2389290, 2389304,	

© 2023 Naveed Z et al. *JAMA Network Open.*

Element/variable	Data source	Definition	Reference
		2389312, 2391600, 2397307, 2403250, 2403269, 2403277, 2403366,2403374, 2403382, 2403412, 2403420, 2403439, 2403447, 2405067, 2406020, 2406039,2408228, 2408236, 2409283, 2409291, 22303140, 45230001, 45230002, 45230003, 45230004, 45230005, 45230006, 45230007, 45230008, 45230009, 45230010, 47450001,47450002, 47450003, 47450004, 47450005, 47450006, 47450007.	
Exposure			
SARS-Cov-2 infection	COVID-19 surveillance case data (ccap_covid_case)	Tested by real-time reverse transcription–polymerase chain reaction (RT-PCR)	British Columbia Centre for Disease Control [creator]. COVID-19 surveillance case data. British Columbia Centre for Disease Control [publisher]
Co-morbidities			
Chronic Kidney Disease	• DAD<br>• MSP<br>• NACRS-HA<br>• NACRS-MOH	ICD-9-CM (MSP): 403.01, 403.11, 403.91, 404.02, 404.03, 404.12, 404.13, 404.92, 404.93, 585.x, 586.x, 588.0, V42.0, V45.1, V56.x<br><br>ICD-10 (DAD/NACRS): I12.0, I13.1, N18.x, N19.x, N25.0, Z49.0 - Z49.2, Z94.0, Z99.2	Quan H, Sundararajan V, Halfon P, Fong A, Burnand B, Luthi J-C, et al. Coding Algorithms for Defining Comorbidities in ICD-9-CM and ICD-10 Administrative Data. Med Care 2005;43:1130–9.
Injection Drug Use	• DAD<br>• MSP<br>• NACRS-HA<br>• NACRS-MOH	Diagnosis age between 11 and 65<br><br>ICD-9 diagnostic codes: starting with 292, 970, 3040, 3041, 3042, 3044, 3045, 3046, 3047, 3048, 3049, 3054, 3055, 3056, 3057, 3059, 6483, 7960, 9621, 9650, 9658, 9663, 9664, 9670, 9684, 9685, 9694, 9696, 9697, 9698, 9699, E8500, V6542; exact code V6542.<br><br>ICD- 10-CA diagnostic codes: starting with F11, F13, F14, F15, F19, R781, R782, T387, T400, T401, T402, T403, T404, T405, T406, T408, T409, T412, T423, T424, T425, T426, T427, T428,	Janjua NZ, Islam N, Kuo M, et al. Identifying injection drug use and estimating population size of people who inject drugs using healthcare administrative datasets. Int J Drug Policy. 2018;55:31-39.

© 2023 Naveed Z et al. *JAMA Network Open.*

Element/variable	Data source	Definition	Reference
		T436, T438, T439, T507; exact codes: R781, R782, T387, T400, T401, T402, T403, T404, T405, T406, T408, T409, T412, T423, T424, T425, T426, T427, T428, T436, T438, T439, T507<br><br>NACRS complaint codes: starting with 751, 753	
Glucocorticoids use	PharmaNet	Dispensation (PharmaNet) of at least one glucocorticoid (oral or injectable) during the year prior to collection date.<br><br>DINPINs corresponding to oral or injectable glucocorticoids: 14834, 14842, 28096, 29300, 30600, 30619, 30627, 30635, 30643, 30651, 30740, 30759, 30767, 213624, 269026, 297151, 460761, 494240, 496219, 499846, 501131, 562408, 562416, 562424, 643122, 664200, 664227, 716715, 716995, 732885, 732893, 751863, 783900, 872520, 872539, 874582, 878618, 878626, 888206, 888214, 888222, 888230, 1934325, 1934333, 1934341, 1977547, 1977555, 1977563, 1999761, 1999869, 2063719, 2063727, 2194120, 2194147, 2194155, 2204266, 2204274, 2219271, 2229540, 2229550, 2230210, 2230211, 2231893, 2231894, 2237835, 2245400, 2245406, 2245407, 2245408, 2260301, 2367947, 2367955, 2387743, 2470632, 16241, 30678, 36137, 2063697, 2063700, 2231895, 2232748, 2232750, 2241229, 2367963, 2367971, 12211, 14893, 15016, 15024, 16438, 16446, 16462, 21679, 21695, 23833, 28185, 29475, 30910, 30929, 30988, 36129, 36366, 93629, 156876, 176834, 210188, 210692, 232092, 232378, 249963, 252417, 271373, 271381,	Based on Laugesen K, Jørgensen JOL, Sørensen HT, Petersen I. Systemic glucocorticoid use in Denmark: a population-based prevalence study. BMJ Open 2017;7:e015237. https://doi.org/10.1136/bmjopen-2016-015237 and Laugesen K, Jørgensen JOL, Petersen I, Sørensen HT. Fifteen-year nationwide trends in systemic glucocorticoid drug use in Denmark. Eur J Endocrinol 2019;181:267–73. https://doi.org/10.1530/EJE-19-0305.

Element/variable	Data source	Definition	Reference
		280437, 285471, 295094, 312770, 349100, 354309, 489158, 501050, 501069, 504416, 508586, 550957, 598194, 607517, 610623, 868426, 868434, 868442, 1946897, 1964070, 1964968, 1964976, 2063190, 2152541, 2194082, 2194090, 2194139, 2230619, 2237044, 2237045, 2237046, 2239534, 2240684, 2240685, 2240687, 2245532, 2250055, 2260298, 2261081, 2279363, 2311267	
Alcohol Misuse	• DAD<br>• MSP<br>• NACRS-HA<br>• NACRS-MOH	ICD-9 diagnostic codes: starting with 291, 303, 3050, 3575, 4255.<br><br>ICD-10-CA: starting with F10, E244, G312, G621, G721, I426, Z502, Z714.	Janjua NZ, Kuo M, Yu A, Alvarez M, Wong S, Cook D, et al. The Population Level Cascade of Care for Hepatitis C in British Columbia, Canada: The BC Hepatitis Testers Cohort (BC-HTC). EBioMedicine 2016;12:189–95.
• Acute myocardial infarction<br>• Asthma<br>• Chronic liver disease<br>• Chronic obstructive pulmonary disease<br>• Depression<br>• Hypertension	Chronic Disease Registry (CDR)	http://www.bccdc.ca/health-professionals/data-reports/chronic-disease-dashboard#Case--Definitions<br><br>Please visit the above link for detailed definitions	British Columbia Ministry of Health [creator]. Chronic Disease Registry. British Columbia Ministry of Health [publisher]. Data Extract. MOH (2020). 2022.
Others			
Material deprivation index	Canadian census (2016)	The Québec Index of Material Deprivation was calculated based on 6-digit postal code of an individual’s residence. This index combines three indicators related to deprivation of goods and conveniences, available by Dissemination Area (DA) in Canadian census data: 1) proportion of persons without high-school diploma 2) ratio of employment to population 3) average income.	Pampalon R, Raymond G. A deprivation index for health and welfare planning in Quebec. Chronic Dis Can 2000;21(3):104-113.

© 2023 Naveed Z et al. *JAMA Network Open.*

Element/variable	Data source	Definition	Reference
Vaccination status	Provincial Immunization Registry	Not vaccinated: Individual has not received any vaccine dose by collection date.<br>Partially vaccinated: At least 14 days have passed since first dose inoculation and collection date.<br>Vaccinated: No less than 14 days have passed since second dose and collection date.	Fibke CD, Joffres Y, Tyson JR, Colijn C, Janjua NZ, Fjell C, et al. Spike Mutation Profiles Associated With SARS-CoV-2 Breakthrough Infections in Delta Emerging and Predominant Time Periods in British Columbia, Canada. Front Public Heal 2022;0:2038. https://doi.org/10.3389/FPUBH.2022.915363.

© 2023 Naveed Z et al. *JAMA Network Open.*

eTable 3. Distribution of Characteristics Among Individuals With Incident Diabetes Cases by COVID-19 Exposure Status

Variables	COVID-19 Negative	COVID-19 Positive	Overall	P-value
	**(N=1864)**	**(N=608)**	**(N=2472)**	
**Sex**				
**Female**	1070 (57.4%)	323 (53.1%)	1393 (56.4%)	0.1816
Male	794 (42.6%)	285 (46.9%)	1079 (43.6%)	
**Age (years)**				
Median (Q1-Q3)	41.5 (34.0 - 51.0)	45.0 (36.0 - 53.0)	42.0 (34.0 - 51.0)	0.0013
**Age group**				
18-39 years	807 (43.3%)	217 (35.7%)	1024 (41.4%)	0.0867
40-59 years	899 (48.2%)	332 (54.6%)	1231 (49.8%)	
60-79 years	154 (8.3%)	57 (9.4%)	211 (8.5%)	
80+ years	4 (0.2%)	2 (0.3%)	6 (0.2%)	
**Health Authority**				
Fraser	972 (52.1%)	354 (58.2%)	1326 (53.6%)	< 0.001
Interior	179 (9.6%)	47 (7.7%)	226 (9.1%)	
Northern	87 (4.7%)	53 (8.7%)	140 (5.7%)	
Vancouver Coastal	404 (21.7%)	136 (22.4%)	540 (21.8%)	
Vancouver Island	222 (11.9%)	18 (3.0%)	240 (9.7%)	
**Follow-up time (days)**				
Median (Q1-Q3)	132 (61.0 - 233)	136 (61.5 - 238)	133 (61.0 - 234)	0.8994
**Acute myocardial infarct, prevalent**	44 (2.4%)	13 (2.1%)	57 (2.3%)	0.9509
**Asthma**	362 (19.4%)	111 (18.3%)	473 (19.1%)	0.8181
**Chronic kidney disease**	74 (4.0%)	22 (3.6%)	96 (3.9%)	0.9269
**Chronic liver disease**	11 (0.6%)	3 (0.5%)	14 (0.6%)	0.9626
**Chronic obstructive pulmonary disease**	41 (2.2%)	8 (1.3%)	49 (2.0%)	0.3979
**Depression**	817 (43.8%)	187 (30.8%)	1004 (40.6%)	< 0.001
**Hypertension**	402 (21.6%)	159 (26.2%)	561 (22.7%)	0.0641
**Alcohol misuse**	142 (7.6%)	49 (8.1%)	191 (7.7%)	0.9393
**Injection drug use**	84 (4.5%)	29 (4.8%)	113 (4.6%)	0.9642
**Glucocorticoids use**	89 (4.8%)	25 (4.1%)	114 (4.6%)	0.7954
**Vaccination status**				
Not vaccinated	1578 (84.7%)	561 (92.3%)	2139 (86.5%)	< 0.001
Partially vaccinated	106 (5.7%)	19 (3.1%)	125 (5.1%)	
Vaccinated	180 (9.7%)	28 (4.6%)	208 (8.4%)	
**Material deprivation index**				
1 (Most privileged)	324 (17.4%)	71 (11.7%)	395 (16.0%)	< 0.001
2	419 (22.5%)	95 (15.6%)	514 (20.8%)	
3	381 (20.4%)	115 (18.9%)	496 (20.1%)	
4	362 (19.4%)	134 (22.0%)	496 (20.1%)	
5 (Most deprived)	286 (15.3%)	171 (28.1%)	457 (18.5%)	
Unknown/Missing	92 (4.9%)	22 (3.6%)	114 (4.6%)	

© 2023 Naveed Z et al. *JAMA Network Open.*

**Material deprivation index (imputed)**				
1 (Most privileged)	341 (18.3%)	71 (11.7%)	412 (16.7%)	< 0.001
2	494 (26.5%)	108 (17.8%)	602 (24.4%)	
3	381 (20.4%)	115 (18.9%)	496 (20.1%)	
4	362 (19.4%)	134 (22.0%)	496 (20.1%)	
5	286 (15.3%)	180 (29.6%)	466 (18.9%)	

© 2023 Naveed Z et al. *JAMA Network Open.*

eTable 4. Overall Adjusted Hazard Ratios for Incident Diabetes From Cox Model by Age, Sex, and Material Deprivation Index

Variable	Adjusted Hazard Ratio (95% CI)	p-value
**COVID-19**	1.17 (1.06 – 1.28)	0.00129
**Vaccination**		
Not vaccinated	Ref	
Partially vaccinated	1.00 (0.83 – 1.20)	0.98726
Vaccinated	0.94 (0.80 – 1.09)	0.3887
**AMI**	1.57 (1.19 – 2.07)	0.00127
**Asthma**	1.28 (1.15 – 1.42)	< 0.001
**CKD**	1.44 (1.16 – 1.78)	0.00105
**Depression**	1.39 (1.27 – 1.51)	< 0.001
**Hypertension**	2.60 (2.32 – 2.92)	< 0.001
**Glucocorticoids**	1.45 (1.25 – 1.69)	< 0.001

© 2023 Naveed Z et al. *JAMA Network Open.*

eTable 5. Adjusted Hazard Ratios for Incident Diabetes in Males

Variable	Adjusted Hazard Ratio (95% CI)	p-value
**COVID-19**	1.22 (1.06 – 1.40)	0.00433
**Age group**		
18-39 years	Ref	
40-59 years	2.88 (2.49 – 3.33)	< 0.001
60-79 years	2.80 (2.13 – 3.67)	< 0.001
80+ years	1.55 (0.40 – 6.07)	0.52905
**Vaccination**		
Not vaccinated	Ref	
Partially vaccinated	1.05 (0.79 – 1.40)	0.73002
Vaccinated	0.84 (0.66 – 1.06)	0.14375
**AMI**	1.69 (1.21 – 2.36)	0.00207
**Asthma**	1.13 (0.95 – 1.34)	0.1621
**CKD**	1.99 (1.50 – 2.64)	< 0.001
**Depression**	1.31 (1.14 – 1.51)	< 0.001
**Hypertension**	2.86 (2.40 – 3.40)	< 0.001
**Glucocorticoids**	1.31 (1.02 – 1.69)	0.03765
**Material deprivation index (imputed)**		
1 (Most privileged)	Ref	
2	1.02 (0.84 – 1.24)	0.84064
3	1.31 (1.07 – 1.59)	0.00827
4	1.25 (1.03 – 1.53)	0.02678
5 (Most deprived)	1.53 (1.25 – 1.86)	< 0.001

© 2023 Naveed Z et al. *JAMA Network Open.*

eTable 6. Adjusted Hazard Ratios for Incident Diabetes in Females From Cox Model Stratified by Material Deprivation Index Score

Variable	Adjusted Hazard Ratio (95% CI)	p-value
**COVID-19**	1.12 (0.99 – 1.27)	0.0804
**Age group**		
18-39 years	Ref	
40-59 years	2.31 (2.05 – 2.61)	<0.001
60-79 years	1.90 (1.51 – 2.38)	<0.001
80+ years	1.69 (0.64 – 4.47)	0.2868
**Vaccination**		
Not vaccinated	Ref	
Partially vaccinated	0.96 (0.76 – 1.22)	0.7656
Vaccinated	1.03 (0.85 – 1.25)	0.7744
**AMI**	1.34 (0.82 – 2.21)	0.2411
**Asthma**	1.39 (1.22 – 1.58)	<0.001
**CKD**	1.04 (0.75 – 1.45)	0.8075
**Depression**	1.44 (1.29 – 1.61)	<0.001
**Hypertension**	2.38 (2.03 – 2.78)	<0.001
**Glucocorticoids**	1.55 (1.29 – 1.88)	<0.001

© 2023 Naveed Z et al. *JAMA Network Open.*

eTable 7. Overall Adjusted Hazard Ratios for Incident Diabetes Among Individuals Hospitalized With COVID-19 vs Test-Negative Control Group From Cox Model Stratified by Sex and Depression Status

Variable	Adjusted Hazard Ratio (95% CI)	p-value
**COVID-19**	2.42 (1.87 – 3.15)	< 0.001
**Age group**		
18-39 years	Ref	
40-59 years	2.50 (2.25 – 2.78)	< 0.001
60-79 years	2.18 (1.78 – 2.68)	< 0.001
80+ years	1.45 (0.60 – 3.50)	0.40954
**Vaccination status**		
Not vaccinated	Ref	
Partially vaccinated	1.04 (0.85 – 1.26)	0.70916
Vaccinated	0.93 (0.79 – 1.09)	0.35465
**AMI**	1.67 (1.23 – 2.28)	0.00113
**Asthma**	1.21 (1.08 – 1.36)	0.00129
**CKD**	1.52 (1.20 – 1.93)	< 0.001
**Hypertension**	2.57 (2.25 – 2.94)	< 0.001
**Glucocorticoids**	1.43 (1.20 – 1.69)	< 0.001
**Material deprivation index (imputed)**		
1 (Most privileged)	Ref	
2	1.14 (0.99 – 1.30)	0.06627
3	1.21 (1.05 – 1.40)	0.00939
4	1.26 (1.09 – 1.46)	0.0021
5 (Most deprived)	1.34 (1.15 – 1.56)	< 0.001

© 2023 Naveed Z et al. *JAMA Network Open.*

eTable 8. Adjusted Hazard Ratios for Incident Diabetes Among Males Hospitalized With COVID-19 vs Test-Negative Control Group

**Variable**	**Adjusted Hazard Ratio (95% CI)**	**p-value**
**COVID-19**	2.84 (2.01 – 4.03)	<0.001
**Age group**		
18-39 years	Ref	
40-59 years	2.88 (2.44 – 3.40)	<0.001
60-79 years	2.96 (2.17 – 4.03)	<0.001
80+ years	0.75 (0.11 – 5.18)	0.7706
**Vaccination status**		
Not vaccinated	Ref	
Partially vaccinated	1.04 (0.77 – 1.42)	0.78509
Vaccinated	0.83 (0.64 – 1.07)	0.14957
**AMI**	1.68 (1.15 – 2.44)	0.00678
**Asthma**	1.07 (0.88 – 1.30)	0.49989
**CKD**	2.10 (1.54 – 2.87)	<0.001
**Depression**	1.31 (1.12 – 1.53)	<0.001
**Hypertension**	2.88 (2.36 – 3.51)	<0.001
**Glucocorticoids**	1.33 (1.00 – 1.77)	0.0465
**Material deprivation index (imputed)**		
1 (Most privileged)	Ref	
2	0.99 (0.80 – 1.23)	0.95122
3	1.26 (1.02 – 1.57)	0.03358
4	1.23 (0.98 – 1.53)	0.06814
5 (Most deprived)	1.36 (1.08 – 1.72)	0.00867

© 2023 Naveed Z et al. *JAMA Network Open.*

eTable 9. Adjusted Hazard Ratios for Incident Diabetes Among Females Hospitalized With COVID-19 vs Test-Negative Controls From Cox Model Stratified by Material Deprivation Index Score

Variable	Adjusted Hazard Ratio (95% CI)	p-value
**COVID-19**	1.94 (1.30 – 2.88)	0.00107
**Age group**		
18-39 years	Ref	
40-59 years	2.23 (1.94 – 2.55)	<0.001
60-79 years	1.77 (1.36 – 2.31)	<0.001
80+ years	2.04 (0.76 – 5.48)	0.15722
**Vaccination status**		
Not vaccinated	Ref	
Partially vaccinated	1.03 (0.80 – 1.33)	0.82028
Vaccinated	1.00 (0.81 – 1.24)	0.96487
**AMI**	1.42 (0.82 – 2.47)	0.2136
**Asthma**	1.34 (1.16 – 1.55)	<0.001
**CKD**	1.10 (0.77 – 1.57)	0.60923
**Depression**	1.65 (1.45 – 1.87)	<0.001
**Hypertension**	2.30 (1.92 – 2.75)	<0.001
**Glucocorticoids**	1.49 (1.20 – 1.85)	<0.001

© 2023 Naveed Z et al. *JAMA Network Open.*

eTable 10. Overall Adjusted Hazard Ratios for Incident Diabetes Among Individuals Admitted to Intensive Care Unit With COVID-19 vs Test-Negative Control Group in Cox Model Stratified by Sex

**Variable**	**Adjusted Hazard Ratio (95% CI)**	**p-value**
**COVID-19**	3.29 (1.98 – 5.48)	< 0.001
**Age group**		
18-39 years	Ref	
40-59 years	2.48 (2.23 – 2.76)	< 0.001
60-79 years	2.23 (1.81 – 2.74)	< 0.001
80+ years	1.48 (0.55 – 3.94)	0.436
**Vaccination status**		
Not vaccinated	Ref	
Partially vaccinated	1.04 (0.85 – 1.27)	0.69945
Vaccinated	0.93 (0.79 – 1.09)	0.37185
**AMI**	1.76 (1.29 – 2.41)	< 0.001
**Asthma**	1.21 (1.08 – 1.37)	0.00133
**CKD**	1.51 (1.19 – 1.92)	< 0.001
**Depression**	1.52 (1.38 – 1.68)	< 0.001
**Hypertension**	2.60 (2.27 – 2.98)	< 0.001
**Glucocorticoids**	1.41 (1.18 – 1.68)	< 0.001
**Material deprivation index (imputed)**		
1 (Most privileged)	Ref	
2	1.14 (0.99 – 1.31)	0.06062
3	1.21 (1.04 – 1.40)	0.01225
4	1.26 (1.09 – 1.46)	0.00201
5 (Most deprived)	1.33 (1.14 – 1.56)	< 0.001

© 2023 Naveed Z et al. *JAMA Network Open.*

eTable 11. Adjusted Hazard Ratios for Incident Diabetes Among Males Admitted to Intensive Care Unit With COVID-19 vs Test-Negative Control Group

**Variable**	**Adjusted Hazard Ratio (95% CI)**	**p-value**
**COVID-19**	3.74 (1.97 – 7.07)	< 0.001
**Age group**		
18-39 years	Ref	
40-59 years	2.84 (2.40 – 3.36)	< 0.001
60-79 years	3.05 (2.23 – 4.18)	< 0.001
80+ years	1.19 (0.18 – 7.74)	0.858
**Vaccination status**		
Not vaccinated	Ref	
Partially vaccinated	1.03 (0.75 – 1.41)	0.86234
Vaccinated	0.83 (0.64 – 1.08)	0.16267
**AMI**	1.81 (1.25 – 2.63)	0.00168
**Asthma**	1.08 (0.89 – 1.32)	0.44302
**CKD**	2.09 (1.52 – 2.88)	< 0.001
**Depression**	1.33 (1.13 – 1.55)	< 0.001
**Hypertension**	2.97 (2.43 – 3.63)	< 0.001
**Glucocorticoids**	1.37 (1.03 – 1.83)	0.03108
**Material deprivation index (imputed)**		
1 (Most privileged)	Ref	
2	1.01 (0.82 – 1.25)	0.90678
3	1.26 (1.01 – 1.57)	0.03766
4	1.22 (0.97 – 1.52)	0.08457
5 (Most deprived)	1.36 (1.07 – 1.72)	0.01133

© 2023 Naveed Z et al. *JAMA Network Open.*

eTable 12. Adjusted Hazard Ratios for Incident Diabetes Among Females Admitted to Intensive Care Unit With COVID-19 vs Test-Negative Control Group From Cox Model Stratified by Material Deprivation Index Score

Variable	Adjusted Hazard Ratio (95% CI)	p-value
**COVID-19**	2.71 (1.18 – 6.18)	0.01818
**Age group**		
18-39 years	Ref	
40-59 years	2.21 (1.93 – 2.54)	<0.001
60-79 years	1.78 (1.36 – 2.33)	<0.001
80+ years	1.82 (0.58 – 5.74)	0.30902
**Vaccination status**		
Not vaccinated	Ref	
Partially vaccinated	1.05 (0.81 – 1.35)	0.73327
Vaccinated	1.00 (0.81 – 1.24)	0.98101
**AMI**	1.42 (0.81 – 2.52)	0.22417
**Asthma**	1.33 (1.15 – 1.54)	<0.001
**CKD**	1.10 (0.76 – 1.59)	0.60829
**Depression**	1.68 (1.48 – 1.92)	<0.001
**Hypertension**	2.29 (1.91 – 2.75)	<0.001
**Glucocorticoids**	1.43 (1.15 – 1.78)	0.0014

© 2023 Naveed Z et al. *JAMA Network Open.*

eTable 13. Estimated Population-Attributable Fraction and 95% Wald CI

Time point (days)	Overall	Males
31	0.0345 (0.0122 - 0.0569)	0.0482 (0.0133 - 0.0831)
100	0.0345 (0.0122 - 0.0568)	0.0481 (0.0133 - 0.0830)
200	0.0344 (0.0121 - 0.0567)	0.0480 (0.0132 - 0.0828)
300	0.0343 (0.0121 - 0.0566)	0.0479 (0.0132 - 0.0826)
400	0.0343 (0.0121 - 0.0565)	0.0478 (0.0132 - 0.0824)
500	0.0342 (0.0121 - 0.0564)	0.0477 (0.0132 - 0.0823)
600	0.0341 (0.0120 - 0.0562)	0.0476 (0.0131 - 0.0820)
700	0.0341 (0.0120 - 0.0561)	0.0475 (0.0131 - 0.0819)

© 2023 Naveed Z et al. *JAMA Network Open.*

eTable 14. Overall Adjusted Hazard Ratio for Incident Insulin-Dependent Diabetes From Cox Model Stratified by Age and Depression Status

Variable	Adjusted Hazard Ratio (95% CI)	p-value
**COVID-19**	1.15 (0.98 – 1.36)	0.08196
**Sex**		
Female	Ref	
Male	1.25 (1.08 – 1.44)	0.00307
**Vaccination**		
Not vaccinated	Ref	
Partially vaccinated	1.10 (0.80 – 1.50)	0.56917
Vaccinated	0.73 (0.55 – 0.97)	0.03143
**AMI**	1.21 (0.70 – 2.08)	0.50022
**Asthma**	1.12 (0.92 – 1.35)	0.26568
**CKD**	1.11 (0.72 – 1.71)	0.63637
**Hypertension**	2.78 (2.27 – 3.41)	< 0.001
**Glucocorticoids**	1.57 (1.20 – 2.05)	<0.001
**MDI**		
1 (Most privileged)	Ref	
2	1.17 (0.92 – 1.48)	0.19934
3	1.45 (1.14 – 1.85)	0.00264
4	1.59 (1.25 – 2.03)	< 0.001
5 (Most deprived)	1.93 (1.52 – 2.44)	< 0.001

© 2023 Naveed Z et al. *JAMA Network Open.*

eTable 15. Overall Adjusted Hazard Ratio for Incident Non–Insulin-Dependent Diabetes From Cox Model Stratified by Sex

Variable	Adjusted Hazard Ratio (95% CI)	p-value
**COVID-19**	1.17 (1.04 – 1.31)	0.00842
**Age group**		
18-39 years	Ref	
40-59 years	2.66 (2.38 – 2.98)	< 0.001
60-79 years	2.13 (1.72 – 2.63)	< 0.001
80+ years	1.44 (0.55 – 3.79)	0.46261
**Vaccination status**		
Not vaccinated	Ref	
Partially vaccinated	0.96 (0.76 – 1.20)	0.69678
Vaccinated	1.04 (0.87 – 1.24)	0.66338
**AMI**	1.86 (1.36 – 2.56)	< 0.001
**Asthma**	1.34 (1.18 – 1.51)	< 0.001
**CKD**	1.59 (1.24 – 2.04)	< 0.001
**Depression**	1.44 (1.29 – 1.60)	< 0.001
**Hypertension**	2.56 (2.23 – 2.94)	< 0.001
**Glucocorticoids**	1.42 (1.18 – 1.70)	< 0.001
**Material deprivation index (imputed)**		
1 (Most privileged)	Ref	
2	1.13 (0.97 – 1.31)	0.11173
3	1.18 (1.01 – 1.38)	0.04134
4	1.19 (1.01 – 1.39)	0.03224
5 (Most deprived)	1.19 (1.01 – 1.40)	0.04194

© 2023 Naveed Z et al. *JAMA Network Open.*

eTable 16. Overall Adjusted Hazard Ratios for Incident Diabetes Among Individuals With More Than 90 d Observation Time From Cox Model Stratified by Sex

Variable	Adjusted Hazard Ratio (95% CI)	p-value
**COVID-19**	1.18 (1.06 - 1.31)	0.00295
**Age group**		
18-39 years	Ref	
40-59 years	2.45 (2.20 - 2.73)	< 0.001
60-79 years	1.91 (1.56 - 2.35)	< 0.001
80+ years	0.99 (0.32 - 3.05)	0.98024
**Vaccination**		
Not vaccinated	Ref	
Partially vaccinated	0.98 (0.79 - 1.23)	0.8952
Vaccinated	0.91 (0.71 - 1.15)	0.42742
**AMI**	1.88 (1.39 - 2.55)	< 0.001
**Asthma**	1.29 (1.15 - 1.45)	< 0.001
**CKD**	1.41 (1.10 - 1.81)	0.00673
**Depression**	1.43 (1.29 - 1.58)	< 0.001
**Hypertension**	2.75 (2.41 - 3.13)	< 0.001
**Glucocorticoids**	1.39 (1.17 - 1.66)	< 0.001
**Material deprivation index (imputed)**		
1 (Most privileged)	Ref	
2	1.16 (1.01 - 1.34)	0.03604
3	1.24 (1.07 - 1.45)	0.00455
4	1.19 (1.02 - 1.39)	0.02471
5 (Most deprived)	1.31 (1.12 - 1.53)	< 0.001

© 2023 Naveed Z et al. *JAMA Network Open.*

eTable 17. Adjusted Hazard Ratios for Incident Diabetes Among Males With More Than 90 d Observation Time From Cox Model

Variable	Adjusted Hazard Ratio (95% CI)	p-value
**COVID-19**	1.24 (1.05 - 1.45)	0.0104
**Age group**		
18-39 years	Ref	
40-59 years	2.61 (2.21 - 3.09)	<0.001
60-79 years	2.26 (1.64 - 3.10)	<0.001
80+ years	0.92 (0.14 - 6.27)	0.9356
**Vaccination**		
Not vaccinated	Ref	
Partially vaccinated	1.04 (0.72 - 1.50)	0.8355
Vaccinated	0.89 (0.61 - 1.30)	0.5369
**AMI**	1.95 (1.34 - 2.82)	<0.001
**Asthma**	1.20 (0.98 - 1.45)	0.0714
**CKD**	1.98 (1.43 - 2.76)	<0.001
**Depression**	1.38 (1.18 - 1.63)	<0.001
**Hypertension**	3.03 (2.49 - 3.69)	<0.001
**Glucocorticoids**	1.22 (0.90 - 1.65)	0.2105
**Material deprivation index (imputed)**		
1 (Most privileged)	Ref	
2	1.02 (0.82 - 1.28)	0.8487
3	1.20 (0.95 - 1.52)	0.1169
4	1.16 (0.92 - 1.46)	0.2158
5 (Most deprived)	1.50 (1.19 - 1.88)	<0.001

© 2023 Naveed Z et al. *JAMA Network Open.*

eTable 18. Adjusted Hazard Ratios for Incident Diabetes Among Females With More Than 90 d Observation Time From Cox Model

Variable	Adjusted Hazard Ratio (95% CI)	p-value
**COVID-19**	1.14 (0.98 - 1.31)	0.08635
**Age group**		
18-39 years	Ref	
40-59 years	2.32 (2.02 - 2.66)	< 0.001
60-79 years	1.74 (1.33 - 2.27)	< 0.001
80+ years	1.10 (0.27 - 4.42)	0.89814
**Vaccination**		
Not vaccinated	Ref	
Partially vaccinated	0.95 (0.71 - 1.26)	0.7199
Vaccinated	0.92 (0.67 - 1.25)	0.59821
**AMI**	1.61 (0.94 - 2.74)	0.08149
**Asthma**	1.37 (1.18 - 1.60)	< 0.001
**CKD**	1.03 (0.70 - 1.51)	0.87286
**Depression**	1.47 (1.29 - 1.67)	< 0.001
**Hypertension**	2.50 (2.10 - 2.98)	< 0.001
**Glucocorticoids**	1.51 (1.22 - 1.88)	< 0.001
**Material deprivation index (imputed)**		
1 (Most privileged)	Ref	
2	1.28 (1.06 - 1.54)	0.00992
3	1.28 (1.05 - 1.56)	0.01458
4	1.22 (1.00 - 1.50)	0.05029
5 (Most deprived)	1.17 (0.95 - 1.45)	0.14557

© 2023 Naveed Z et al. *JAMA Network Open.*

eTable 19. Overall Adjusted Hazard Ratios for Incident Diabetes in Unvaccinated Subpopulation From Cox Model Stratified by Sex, Age, Material Deprivation Index Score

Variable	Adjusted Hazard Ratio (95% CI)	p-value
**COVID-19**	1.18 (1.07 - 1.30)	0.00118
**AMI**	1.55 (1.15 - 2.10)	0.00432
**Asthma**	1.28 (1.14 - 1.43)	< 0.001
**CKD**	1.49 (1.18 - 1.88)	< 0.001
**Depression**	1.40 (1.27 - 1.54)	< 0.001
**Hypertension**	2.72 (2.40 - 3.08)	< 0.001
**Glucocorticoids**	1.45 (1.23 - 1.71)	< 0.001

© 2023 Naveed Z et al. *JAMA Network Open.*

eTable 20. Overall Adjusted Hazard Ratios for Incident Diabetes in Partially Vaccinated Subpopulation

Variable	Adjusted Hazard Ratio (95% CI)	p-value
**COVID-19**	0.86 (0.53 - 1.41)	0.56085
**Sex**		
Female	Ref	
Male	1.04 (0.72 - 1.49)	0.84906
**Age group**		
18-39 years	Ref	
40-59 years	2.28 (1.46 - 3.57)	< 0.001
60-79 years	1.89 (0.90 - 3.99)	0.09384
80+ years	5.59 (1.4 - 22.27)	0.01472
**AMI**	1.52 (0.57 - 4.02)	0.40088
**Asthma**	1.00 (0.62 - 1.61)	0.99467
**CKD**	1.07 (0.47 - 2.45)	0.86834
**Depression**	1.45 (0.98 - 2.14)	0.06319
**Hypertension**	2.93 (1.79 - 4.82)	< 0.001
**Glucocorticoids**	2.10 (1.22 - 3.63)	0.00756
**Material deprivation index (imputed)**		
1 (Most privileged)	Ref	
2	1.43 (0.81 - 2.52)	0.21176
3	1.53 (0.83 - 2.80)	0.1733
4	1.25 (0.67 - 2.34)	0.48762
5 (Most deprived)	1.42 (0.75 - 2.67)	0.28138

© 2023 Naveed Z et al. *JAMA Network Open.*

eTable 21. Overall Adjusted Hazard Ratios for Incident Diabetes in Vaccinated Subpopulation

Variable	Adjusted Hazard Ratio (95% CI)	p-value
**COVID-19**	1.21 (0.80 - 1.81)	0.36648
**Sex**		
Female	Ref	
Male	0.81 (0.61 - 1.08)	0.15133
**Age group***		
18-39 years	Ref	
40-59 years	3.03 (2.22 - 4.12)	< 0.001
60+ years	2.04 (1.08 - 3.85)	0.028
**AMI**	2.11 (0.87 - 5.14)	0.10054
**Asthma**	1.42 (1.01 – 2.00)	0.04628
**CKD**	1.13 (0.48 - 2.61)	0.78407
**Depression**	1.27 (0.95 - 1.69)	0.11391
**Hypertension**	1.49 (0.97 - 2.28)	0.07097
**Glucocorticoids**	1.07 (0.60 - 1.89)	0.82835
**Material deprivation index (imputed)**		
1 (Most privileged)	Ref	
2	1.53 (0.98 - 2.39)	0.0627
3	1.82 (1.14 - 2.90)	0.01163
4	2.22 (1.40 - 3.53)	< 0.001
5 (Most deprived)	2.10 (1.27 - 3.47)	0.00396

*Due to sample size the last two categories of age were collapsed

© 2023 Naveed Z et al. *JAMA Network Open.*
