# Supplementary material for: Association of COVID-19 Infection With Incident Diabetes
Source: JAMA Netw Open. 2023 Apr 18;6(4):e238866. doi: 10.1001/jamanetworkopen.2023.8866 (PMC10114057; doi:10.1001/jamanetworkopen.2023.8866)
Supplement: Supplement 2. — Data Sharing Statement [file jamanetwopen-e238866-s002.pdf]

Data Sharing Statement

Naveed. Association of COVID-19 Infection With Incident Diabetes. *JAMA Netw Open*. Published online April 18, 2023. doi:10.1001/jamanetworkopen.2023.8866

Data
Data available: No

Additional Information
Explanation for why data not available: The study is based on data contained in various provincial registries and databases. Access to data could be requested through the BC Centre for Disease Control Institutional Data Access for researchers who meet the criteria for access to confidential data. Requests for the data may be sent to datarequest@bccdc.ca.

© 2023 Naveed Z et al. *JAMA Network Open*.
